# Meta-analysis of individual registry results enhances international registry collaboration

**DOI:** 10.1080/17453674.2018.1454383

**Published:** 2018-03-28

**Authors:** Elizabeth W Paxton, Maziar Mohaddes, Inari Laaksonen, Michelle Lorimer, Stephen E Graves, Henrik Malchau, Robert S Namba, John Kärrholm, Ola Rolfson, Guy Cafri

**Affiliations:** a Kaiser Permanente, San Diego, CA, USA;; b Institute of Clinical Sciences, Department of Orthopaedics, Sahlgrenska Academy, University of Gothenburg, Gothenburg, Sweden;; c Swedish Hip Arthroplasty Register, Gothenburg, Sweden;; d Turku University Hospital, Turku, Finland;; e South Australia Health & Medical Research Institute, Adelaide, SA, Australia;; f University of South Australia, Adelaide, SA, Australia;; g Harvard Medical School, Boston, MA, USA;; h Southern California Permanente Medical Group, Irvine, CA, USA

## Abstract

**Background and purpose — Although common in medical research, meta-analysis has not been widely adopted in registry collaborations. A meta-analytic approach in which each registry conducts a standardized analysis on its own data followed by a meta-analysis to calculate a weighted average of the estimates allows collaboration without sharing patient-level data. The value of meta-analysis as an alternative to individual patient data analysis is illustrated in this study by comparing the risk of revision of porous tantalum cups versus other uncemented cups in primary total hip arthroplasties from Sweden, Australia, and a US registry (2003–2015).**

**Patients and methods — For both individual patient data analysis and meta-analysis approaches a Cox proportional hazard model was fit for time to revision, comparing porous tantalum (n = 23,201) with other uncemented cups (n = 128,321). Covariates included age, sex, diagnosis, head size, and stem fixation. In the meta-analysis approach, treatment effect size (i.e., Cox model hazard ratio) was calculated within each registry and a weighted average for the individual registries’ estimates was calculated.**

**Results — Patient-level data analysis and meta-analytic approaches yielded the same results with the porous tantalum cups having a higher risk of revision than other uncemented cups (HR (95% CI) 1.6 (1.4–1.7) and HR (95% CI) 1.5 (1.4–1.7), respectively). Adding the US cohort to the meta-analysis led to greater generalizability, increased precision of the treatment effect, and similar findings (HR (95% CI) 1.6 (1.4–1.7)) with increased risk of porous tantalum cups.**

**Interpretation — The meta-analytic technique is a viable option to address privacy, security, and data ownership concerns allowing more expansive registry collaboration, greater generalizability, and increased precision of treatment effects.**

Orthopedic registries play a critical role in the identification of clinical best practices, outcome assessment, and device surveillance (Herberts and Malchau 1999, 2000, Graves 2010, Paxton et al. 2012, 2013). Collaborations among registries provide additional opportunities to increase statistical power, improve generalizability, and to examine variation in clinical practices and outcomes between countries (Havelin et al. 2009). Previously, registries have collaborated by sending de-identified standardized patient-level data to a centralized database and conducting statistical analyses based on the pooled individual patient-level data (Dale et al. 2012, Bergh et al. 2014, Wangen et al. 2017). Although analysis of individual patient data is an ideal approach, many registries cannot share even de-identified patient level data due to privacy, security, and data ownership regulations (Sedrakyan et al. 2014). One alternative is to collect effect sizes from similarly designed registry studies and perform a meta-analysis.

Meta-analysis is a common approach used in medical research to summarize the findings of several independent studies into a single estimate of the treatment effect (Hedges and Vevea 1998, Borenstein et al. 2009). Well-designed meta-analyses can provide more precise estimates of the treatment effects of individual studies, resulting in a higher level of scientific evidence than individual clinical studies. Typically, meta-analysis consists of weighted averages of the independent study effect sizes, which can be combined using either a fixed- or random-effects model. In a fixed-effect model it is assumed that there is one true effect size common to all studies in the meta-analysis and the combined effect estimates this parameter. In a random-effect model, the effect size is assumed to vary from study to study due to study-specific differences and the combined effect is an estimate of the mean of this distribution. The choice of which model to apply should be based on the perceived process that generates the data as well as the type of inferences desired (Hedges and Vevea 1998, Borenstein et al. 2009). While random-effect models are appealing since studies may differ (e.g., in patient characteristics, inclusion criteria, and methods) and we often want to generalize beyond data of the studies included in a meta-analysis, standardizing the design and analysis in each study can mitigate the impact of study-specific variation. Further, when there are a very small number of studies, the variation among the effects will be estimated imprecisely and this can adversely impact inferences. In these cases, the fixed-effects model is an alternative (Borenstein et al. 2009) despite having more restricted inferences than the random-effects model.

Although meta-analysis of independent studies is frequently used in medical research, often there are a limited number of available studies with a comparable design/analysis that make use of orthopedic registry data (e.g., examining similar treatments, conditioning on the same covariates). As a result, alternative methods for data sharing and collaboration must be considered. One alternative approach is to use a standardized design and analysis that each registry applies on its own registry data, in turn generating model results (e.g., hazard ratios) that can be meta-analyzed across the registries. In this meta-analysis of standardized studies approach, the estimate of the effect is calculated within each registry followed by an averaging of the estimate across the registries for a combined result.

Recently, a variant of meta-analysis of standardized studies was adopted in which aggregate-level survival curve data were meta-analyzed (Banerjee et al. 2014, Cafri et al. 2015). Despite the potential benefits of meta-analysis of standardized studies and prior successful implementations (Allepuz et al. 2014, Graves et al. 2014, Namba et al. 2014, Paxton et al. 2014, Sedrakyan et al. 2014), the method is not well understood or widely adopted among orthopedic registries. One way to motivate this approach is through a comparison of results obtained by meta-analysis of standardized studies and analysis of individual patient data.

Therefore, the purpose of this study is to illustrate the value of meta-analysis of standardized studies as an alternative to analysis of individual patient data. The example in this study compares the risk of revision of porous tantalum cups versus other uncemented cups in primary total hip arthroplasties using data from Sweden, Australia, and a US cohort.

## Patients and methods

Primary total hip replacements with a porous tantalum design cup and other uncemented cups implanted between 2003 and 2015 were identified using the Australian Orthopedic Association National Joint Replacement Registry (AOANJRR), the Swedish Hip Arthroplasty Registry (SHAR), and the Kaiser Permanente Total Joint Registry. The capture rate of these registries exceeds 95% and loss to follow-up is less than 8% over the study period. Validation and quality-control methods of these registries have been published previously (Soderman et al. 2000, Paxton et al. 2012, 2013, Australian Orthopaedic Association 2016). The study sample was restricted to metal on highly crosslinked polyethylene primary THAs. Patient-level data were combined from AOANJRR and SHAR into a centralized database to compare the analysis of individual patient data with meta-analysis of standardized studies. De-identified patient demographics, implant characteristics, and reasons for revisions were extracted from each registry. The US registry was prohibited from providing case-level data and therefore only provided summary-level data for the meta-analytic approach.

### Statistics

The primary objective of this article is a comparison of analysis of individual patient data with meta-analysis of standardized studies. Ideally such a comparison is undertaken when the estimate in both approaches is the same. Beyond comparability, our proposed statistical analyses address confounding due to measured prosthesis/patient characteristics and static study characteristics (e.g., average age), as well as dependency among observations on the response due to the nesting of observations within a registry. For both individual patient data analysis and meta-analysis approaches a Cox proportional hazard model was fit for the endpoint of time to revision (for any component and reason), the treatment effect compares porous tantalum cups with other uncemented cups, and covariates to include age (continuous), sex, diagnosis, (osteoarthritis, rheumatoid arthritis, osteo-/avascular necrosis, hip dysplasia, other), head size (28, 32, > 32), and stem fixation (cemented, uncemented). Missing data were listwise deleted. There were small amounts of missing data on age (n = 27) and sex (n = 32), but more substantial missing data on whether cement was used on the stem (n = 3,438). In the meta-analysis approach a treatment effect size (i.e., hazard ratio from the Cox model) was calculated within each registry, therefore there is no dependency of observations on the response within registry and no confounding due to static study-level characteristics. To address these issues in the individual patient data approach we stratified on study (Glidden and Vittinghoff 2004, Sjölander et al. 2013), which also leads to an estimate comparable to the one obtained from meta-analysis of standardized studies because both approaches allow for each study to have its own distinct baseline hazard. There are some alternatives to stratification that might be considered for analysis of individual patient data, but none of these provide estimates that are more comparable to the meta-analysis approach than the stratification approach adopted. One alternative is a between-within frailty model (Sjölander et al. 2013), but this introduces more parametric assumptions (i.e., distribution of frailties and functional form of cluster effects) than stratification. Another option is use of cluster robust standard errors (Lee et al. 1992), but this does not address study-level confounding. Lastly, inclusion of a dummy indicator for study is possible, but this invokes a proportional hazard assumption for the cluster effect.

Calculating the average treatment effect from a fixed-effect model for a meta-analysis of standardized studies is straightforward (Hedges and Olkin 1985). For each study (i = 1,2,…,k) we estimate a log hazard ratio from a Cox model,
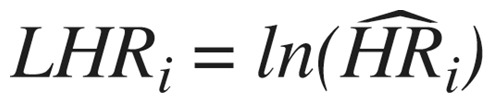
 The variance of this estimate is denoted by 
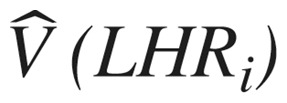
 and a weight is constructed by taking the inverse of this quantity, 
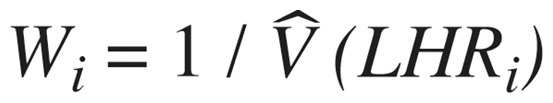
 The average treatment effect across studies is then estimated using a weighted mean, 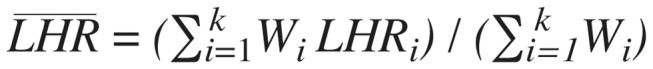 The variance of this mean is then 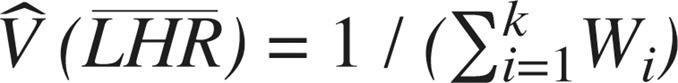 and the standard error is 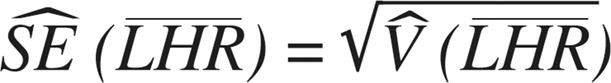 Normal theory confidence intervals (95%) can be calculated in the conventional way: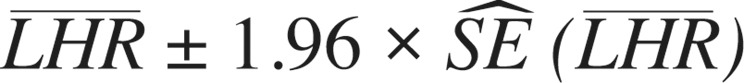 Point estimates and interval endpoints are exponentiated for improved interpretability (e.g.,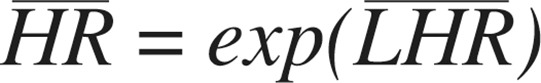). A 2-tailed p-value is based on 
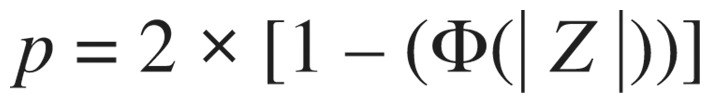
 where *p* is the standard normal cumulative distribution and 
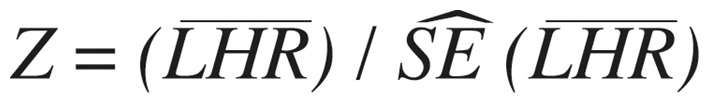


### Ethics, funding, and potential conflicts of interest

Approval from the Institutional Review Board was obtained prior to the start of this study. IRB #5488 approved on August 27, 2009. The study was also approved by the Regional Ethical Review Board in Gothenburg, Sweden (entry number 669-16). There is no funding. There are no potential conflicts of interest.

## Results

**Figure 1.**
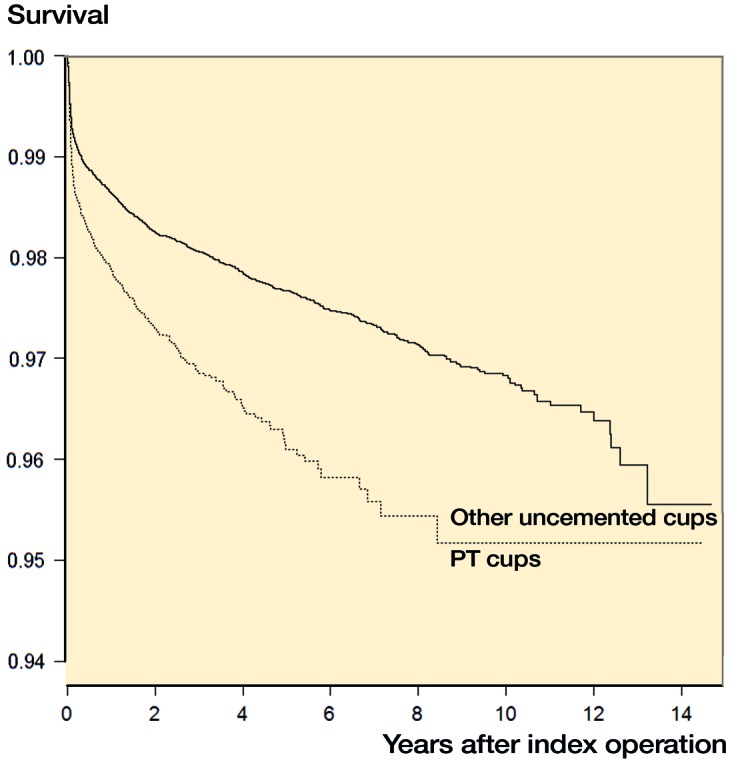
US cohort porous tantalum versus other uncemented cup survival. **Number at risk**:

The porous tantalum group consisted of 2,796 from SHAR, 7,317 from the AOANJRR, and 13,088 from the US registry. Other uncemented cups consisted of 13,156 from SHAR, 70,440 from the AOANJRR, and 44,725 from the US registry. Patient, implant, and fixation, and outcomes of porous tantalum versus other uncemented cup has been reported on SHAR and AOANJRR cohorts (Laaksonen et al. 2018). Therefore, descriptive statistics and Kaplan–Meier survival focus on solely on the US cohort. The US cohort (Table 1) was similar to SHAR and AOANJRR in age, sex, diagnosis, and follow-up. Tables 2 and 3 display cup designs, reasons for revisions, and type of revision for the US cohort. The unadjusted survival of the cups suggested a difference among the groups (Figure 1.). The US cohort also had similar covariate adjusted results to SHAR and AOANJRR, with a higher risk of revision for the porous tantalum group (HR =1.6 (95% CI 1.4–1.8)). When limiting the revision endpoint to cup revisions (i.e., alone or in combination with any other components), covariate adjusted results in the US cohort also indicated a higher risk of revision for the porous tantalum group (HR =1.4 (95% CI 1.0–1.8). The comparison of analysis of patient-level data and the meta-analytic approaches for SHAR and AOANJRR resulted in similar findings, with porous tantalum having a higher risk of revision than other uncemented cups in the covariate-adjusted models (Table 4). When the US cohort’s data was added to Swedish and Australian data, results further indicated a higher risk of revision for porous tantalum cups versus all other uncemented cups (HR =1.6 (95% CI 1.4–1.7). The addition of the US registry data results in greater generalizability and increased precision of estimates (Table 4).

**Table 1.** US cohort cup designs

Cup design	n (%)
Porous tantalum	
Continuum	9,740 (17)
Trabecular metal (shell)	3,348 (5)
Other uncemented	
Trident	1,107 (2)
Pinnacle	34,350 (59)
Trilogy	2,203 (4)
Reflection	7,065 (13)
Allofit	NA
Exceed	NA

**Table 2.** US cohort descriptive data

Factor	Porous tantalum cups	Other uncemented cups
n (%)	13,088 (23)	44,725 (77)
Mean age (range)	66 (16–97)	67 (13–98)
Male (%)	5,447 (42)	18,369 (41)
Right side (%)	7,139 (55)	24,412 (55)
Diagnosis, n (%)		
OA	12,000 (92)	40,987 (92)
RA	203 (2)	656 (1)
Femoral neck fracture	NA NA	NA NA
Dysplasia	249 (2)	690 (2)
Osteonecrosis	524 (4)	2,059 (5)
Other	112 (1)	333 (1)
Follow up, years (range)	2.8 (0–14)	4.6 (0–15)
Uncemented stem, n (%)	12,712 (97)	41,328 (92)
Femoral head size, mm, n (%)		
28	430 (3)	6,186 (14)
32	5,403 (41)	16,190 (36)
32	7,255 (55)	22,349 (50)

**Table 3.** Reasons for revision and type of revision in US cohort ^a^

Factor	Porous tantalum cups n (%)	Other uncemented cups n (%)
Revised	374 (3)	979 (2)
Reason for revision		
Infection	76 (20)	233 (24)
Fracture	11 (3)	47 (5)
Instability	118 (32)	337 (34)
Loosening	51 (14)	108 (11)
Others	118 (32)	254 (26)
Type of revision		
Cup + stem exchange	13 (3)	47 (5)
Stem exchange	121 (32)	209 (21)
Cup exchange	47 (13)	154 (16)
Liner +/– head exchange	107 (29)	346 (35)
Femoral head exchange	8 (2)	32 (3)
Extraction	3 (1)	4 (0)
Others	75 (20)	187 (19)

**^a^**“Revised” and “Reason for revision” entries correspond to validated revision information. “Type of revision” is based on surgeon self-reported procedure.

**Table 4.** Comparison of traditional and meta-analytic approaches

Approach	HR (95% CI)	b	SE	p-value
Sweden (SHAR)	1.45 (1.14–1.85)	0.37	0.124	0.003
Australia (AOANJRR)	1.57 (1.38–1.79)	0.45	0.066	< 0.001
US cohort	1.60 (1.41–1.80)	0.47	0.063	< 0.001
Individual patient data				
(AOANJRR and SHAR)	1.56 (1.39–1.75)	0.44	0.059	< 0.001
Meta-analysis				
(AOANJRR and SHAR)	1.54 (1.38–1.73)	0.44	0.058	< 0.001
(AOANJRR, SHAR, US Cohort)	1.57 (1.44–1.70)	0.45	0.043	< 0.001

**Table:** 

Year	0	2	4	6	8	10	12	14
PT cups	13,088	7,178	3,285	1,051	446	109	6	1
Other cups	44,725	31,669	22,035	14,010	8,628	4,220	1,083	42

## Discussion

This study has important implications for future international registry collaborations. First, similar results were obtained with analysis of individual patient data and meta-analysis of standardized studies using data from the same registries. This is because both approaches are comparable: they estimate the average cup effect by allowing each registry to have its own distinct baseline hazard. Although an analysis of individualized patient data provides more flexibility since the analyses do not need to be pre-specified, the meta-analytic approach allows each registry to control how data are analyzed and shared. Additional benefits of the meta-analytic approach are minimizing privacy and security issues to enhance international registry collaborations, which are critical for increased statistical power and generalizability in detection of implant problems, identification of variation in clinical care and outcomes, and for conducting comparative effectiveness studies. A limitation of the fixed-model approach applied in this study is the more restricted inferences than in a random-effects model. This method also assigns weights based on individual study variance, resulting in more weight to larger registry studies.

Both individual patient data analysis and meta-analysis of standardized studies are characterized by some important assumptions in their implementation in this article, among which are: (1) proportional hazards assumption for the treatment variable and covariates, (2) correct functional form for continuous variables (i.e., age effect is linear) and (3) no interactions among the explanatory variables. Although not explored in this article, alternative statistical models could be adopted that mitigate or eliminate the impact of these assumptions. For instance, a time-dependent treatment effect could be modeled if the treatment effect varied over time.

In addition to contributing to registry methodological advancements, this study also has clinical implications. The AOANJRR and SHAR study reported porous tantalum cups having a higher risk of revision than other uncemented cups (Laaksonen et al. 2018). This study is the first to confirm these findings in a large US cohort. Our study also found that when focusing on cup revisions (with or without revision to other components), the porous tantalum group still had a higher risk of revision than other uncemented cups. This finding differs from a recent UK study focused on a single manufacturer as the control group whereas our study used all uncemented cups as the comparison group (Matharu et al. 2018). Differences in the study comparison groups, design, statistical analyses, and populations most likely explain the differences in findings.

Although the porous tantalum cup may be effective in revision THAs or complicated primaries, the consistent findings of increased risk across 3 countries suggests the need to further investigate the use of this cup in primary THA procedures. The strengths of this study include the inclusion of high-quality data from 3 different countries allowing assessment of generalizability of the findings. Limitations of this study include the intermediate term follow-up in assessing risk of revision in porous tantalum versus other uncemented cups. However, early results seem to indicate a difference in risk of revision and should become further evaluated in longer term studies. Porous tantalum cups may also be used in more complex cases, which could potentially account for the difference in risk of revision. Future studies including radiographic analyses may shed light on complexity of the total hip arthroplasty within these groups.

In summary, meta-analysis provides an opportunity to collaborate across registries when patient-level data sharing is not feasible. Combining data from multiple registries can enhance precision of estimated effects but is less flexible for conducting statistical analyses. While patient-level data analysis is preferable, meta-analyses provides an attractive alternative option.

EP: conception of study, interpretation of data, and manuscript preparation. MM, IL, SG, HM, RN, JK, OR: interpretation of data and manuscript preparation. ML, GC: statistical analyses, interpretation of data, and manuscript preparation.

*Acta* thanks Anne Lübbeke and other anonymous reviewers for help with peer review of this study.
